# A Model of Perinatal Ischemic Stroke in the Rat: 20 Years Already and What Lessons?

**DOI:** 10.3389/fneur.2018.00650

**Published:** 2018-08-07

**Authors:** Christiane Charriaut-Marlangue, Olivier Baud

**Affiliations:** ^1^INSERM U1141 PROTECT, Université Paris Diderot, Sorbonne Paris Cité, Hôpital Robert Debré, Paris, France; ^2^Division of Neonatology and Pediatric Intensive Care, Children's Hospital, Geneva University Hospitals (HUG), University of Geneva, Geneva, Switzerland

**Keywords:** neonatal ischemia, reperfusion, collaterals, nitric oxide, oxidative stress, microglia, cell death, sexual dimorphism

## Abstract

Neonatal hypoxia-ischemia (HI) and ischemia are a common cause of neonatal brain injury resulting in cerebral palsy with subsequent learning disabilities and epilepsy. Recent data suggest a higher incidence of focal ischemia-reperfusion located in the middle cerebral artery (MCA) territory in near-term and newborn babies. Pre-clinical studies in the field of cerebral palsy research used, and still today, the classical HI model in the P7 rat originally described by Rice et al. (1). At the end of the 90s, we designed a new model of focal ischemia in the P7 rat to explore the short and long-term pathophysiology of neonatal arterial ischemic stroke, particularly the phenomenon of reperfusion injury and its sequelae (reported in 1998). Cerebral blood-flow and cell death/damage correlates have been fully characterized. Pharmacologic manipulations have been applied to the model to test therapeutic targets. The model has proven useful for the study of seizure occurrence, a clinical hallmark for neonatal ischemia in babies. Main pre-clinical findings obtained within these 20 last years are discussed associated to clinical pattern of neonatal brain damage.

## Introduction

Neonatal arterial ischemic stroke (NAIS) is defined as a focal disruption of cerebral blood flow mainly affecting the middle cerebral arterial (MCA) territory and diagnosed after radiological (2) and/or pathological evidence in live newborns, without knowing the exact timing of stroke onset. Epidemiologic data suggest that the incidence of NAIS is between 5 and 43 per 100,000 live births and remains stable over time (3). Groups at risk for NAIS are newborns (the first 28 days of life), especially full-term infants, and older children with sickle cell anemia, or congenital heart deficiencies (4). NAIS is a perinatal brain injury not associated with the typical intrapartum risk factors associated with hypoxic–ischemic (HI) encephalopathy (5). HI affects 1.5–3 and up to 6 per 1,000 livebirths in developed and developing countries, respectively (6). With a similar incidence both NAIS and HI newborns develop what is called neonatal cerebral palsy and exhibit symptoms including motor, cognitive and/or behavioral disabilities, and seizures. Seizures represent the clinical outcome that triggers assessment in neonates with stroke (7). After the occurrence of seizures during a neonatal stroke, there is a nearly 3-fold increased risk of later epilepsy (8). Currently, there is no specific treatment for neonatal stroke. The current strategy is mainly based on supportive care, including the management of neonatal seizures to avoid additional brain injury.

Relevant animal models are considered as of crucial importance to explore mechanisms underlying the disease they are supposed to replicate and to assess the safety and efficacy of treatments. In 1998, we reported a new model of NAIS in the rat brain and this review summarizes most of the lines of research and therapeutic strategies in lights with the hypoxic-ischemic (HI) model of Rice-Vannucci, which represents the most common model of HI encephalopathy investigated since 1981 (1) and compared to the endovascular ischemic model.

## The choice of the model, cerebral infarction and seizure incidence (Table 1)

**Figure 1:**
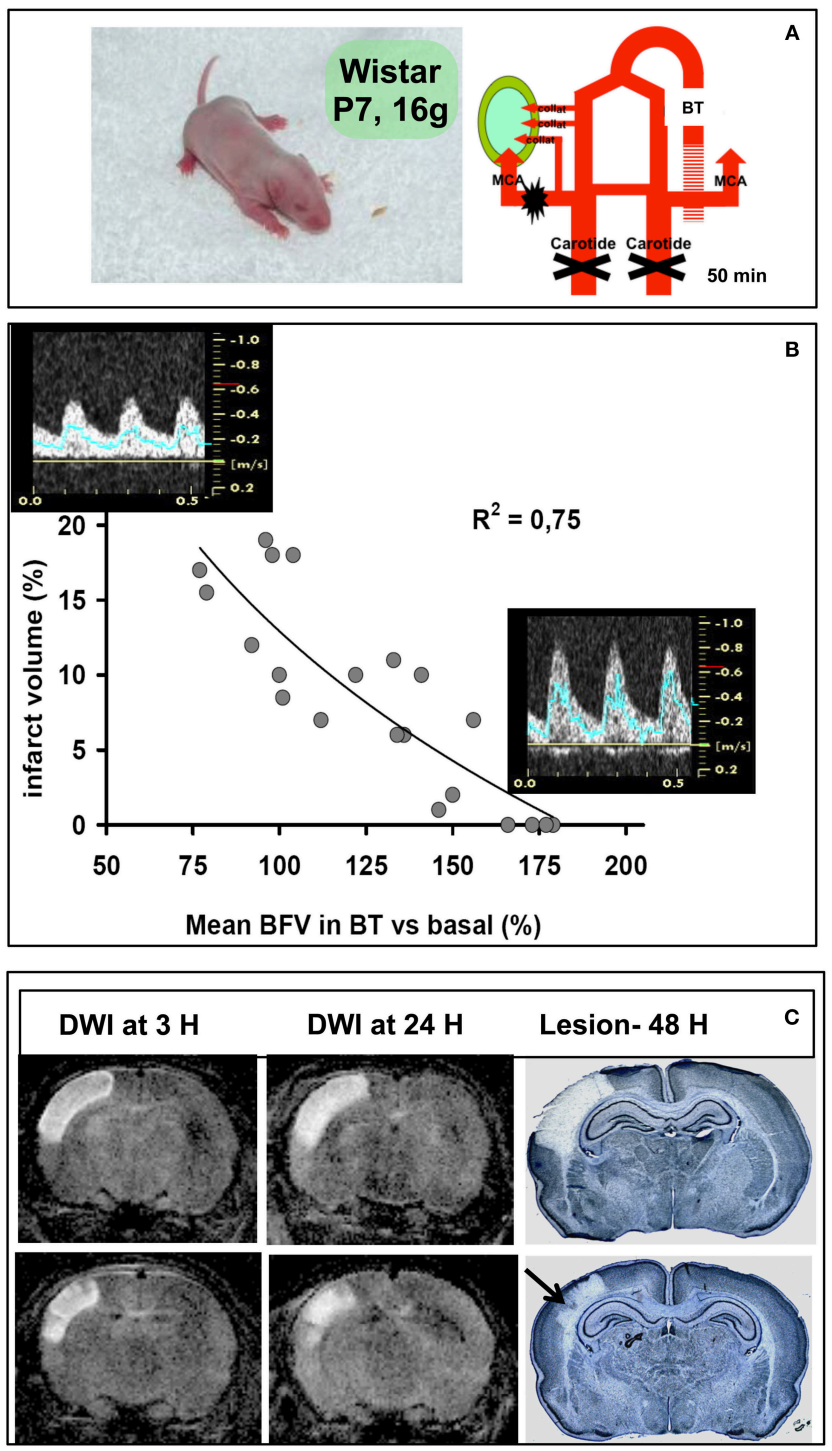
Ischemia-reperfusion in the P7 rat pup. **(A)** Schematic diagram of the model (left) with electrocoagulation of the left MCA and transient (50 min) left and right CCA occlusion (right). The lesion is indicated with the green area, which depends from the presence (or not) of collaterals. **(B)** Regression between percentage of the ischemic lesion volume and percentage of mean blood-flow velocity (mBFV) measured in the basilar trunk (BT). Images of pulsed Doppler signals obtained in animals with a large lesion (left) compared to animals with a small lesion and/or without a lesion (right). **(C)** Diffusion-weighted MRI images (DWI) obtained at 3 and 24 h of reperfusion, and subsequent pale lesion observed on cresyl violet-stained coronal sections.

At the end of the “90s” our goal was to investigate short- and long-term pathophysiology of NAIS, through the phenomenon of reperfusion injury and its consequence in the developing brain. At this time, two labs reported transient focal ischemia in the postnatal 14–18 days (P14–P18) rat (9) and P10 rat (10), the first model being considered as a juvenile stroke model. Using studies regarding neurodevelopmental parallels across species, there is now great evidence that models using P7–P10 rat or mouse can be more characteristic of injuries observed in near- and full-term babies (11). Our model was developed in the P7 rat by combining permanent left MCA electrocoagulation (pMCAo) with transient left common carotid artery (CCA) occlusion (12) (Figure 1A), which represents a model of focal ischemia with arterial reperfusion—but not complete reperfusion—because of the pMCAo. This ischemic model can be paralleled with the unilateral carotid ligation plus 8% FiO2 model, developed by Rice and Vannucci, which should be better considered as a HI model in which there is reoxygenation at return to 21% O_2_ (8). The mortality rate in our ischemia-reperfusion (IR) model is not greater than that observed in the HI model, and lesser than that reported in the endovascular model (9). However, the endovascular model was reported in the P7 rat (13, 14) with not too much mortality. Over all these years, our model has progressed according to the anesthetic used (chloral hydrate vs. isoflurane) leading to transient occlusion of one (IR/1 model) or both (IR/2 model) CCA(s) (15). This model can be applied to animals at a various stage by inducing a longer occlusion of the two CCA as reported in the P15 rat (16, 17).

**Table 1:** An overview of hypoxic-ischemic and ischemic models in immature rats.

**Model**	**Rat strain**	**Age**	**Experimental procedure**	**Histological changes and outcomes**	**References**
Hypoxia-Ischemia (HI)	SD or Wistar	P6-P7	CCAL (left or right) + hypoxia (FiO_2_ 8%)	Damage in the IL hemisphere (cortex, WM, striatum, hippocampus, thalamus, basal ganglia)—Scoring of the lesion (MAP2 loss: 86% at 24 h) Columnar cell death in the cortex, necrosis and apoptosis—Sexual dimorphism Cystic cavitation at 2 weeks	(1, 30–32)
Hypoxia-Ischemia (HI)	Wistar	P12	Right CCAL + hypoxia (FiO_2_ 8%)	Seizure occurrence—damage [48–80%]	(26)
Ischemia-reperfusion (IR)	Wistar	P7	pMCAO + transient CCAo (left CCA or both CCA)	Damage in the IL hemisphere (cortex, WM, head of the caudate putamen)—Lesion volume at 48 h of recovery: 17 ± 10% of the IL hemisphere. Mortality: between 5 and 10% Columnar cell death in the cortex and apoptosis—Sexual dimorphism Bursts and seizures occurrence Cystic cavitation at 1 month	(12, 15, 19, 20, 22, 24)
Ischemia-reperfusion	SD	P12	pMCAO + transient left CCAo	Damage in the IL hemisphere—both apoptosis and autophagy in different neurons	(62, 63)
Ischemia-reperfusion	SD	P7 P10	Transient MCAo (tfMCAo) (endovascular model)	Damage in the IL hemisphere (cortex and subcortical areas- caudate nucleus)—Lesion volume at 24 h of recovery: 34 ± 10% of the IL hemisphere. Mortality: <15%	(13, 14)

*SD, Sprague-Dawley; CCAL, common carotid artery ligation; CCAo, CCA occlusion; pMCAo, permanent middle cerebral artery occlusion; tfMCAo, transient filament MCAo; IL, ipsilateral; WM, white matter*.

Infarct lesion (as evaluated with cresyl violet and/or TTC staining, and MRI) and cell death (as evaluated with the TUNEL assay) at short term (24–48 h post stroke) in the P7 rat are observed in the MCA vascular territory [i.e., cortex and subcortical areas including the white matter (18)] in both models (IR and endovascular). The lesion volume is lesser in our model (mean around 15–18% of the ipsilateral hemisphere) (19, 20) than that measured in the endovascular (mean around 35% of the ipsilateral hemisphere) (14) and in the HI (score of the lesion) model. Using MRI in P10 rat pups, it was reported that injury after HI is more generalized and greater in cortical regions as compared to stroke (endovascular model) in which injury was confined to the vascular territory of the MCA (21). During the next 2 weeks, the infarct evolves into a smooth-walled cavity (12, 22), and 3 months later a substantial cortical zone (at the level of the MCA territory) disappears (12). The differences in the distribution of injury between HI (cortex and deeper gray nuclei—basal ganglia and thalamus) and stroke (MCA territory) indicate that evident greater damage at long-term in HI may likely to result in more important behavioral outcomes as the contralateral side may in some cases be involved in the injury pattern (21).

Ideal stroke models, namely in the rodents, do not entirely reflect the human physiology which is diverse and heterogeneous (focal or global ischemia; left or right side; delays between stroke onset and symptom onset) with diverse risk factors and clinical presentations. However, rodents have several advantages (small number of animals; more or less standardization in the lesion size; same delay to develop the damage; good survival rate and access to cerebral tissue) for investigating the underlying mechanisms associated with factors involved in the pathogenesis of stroke.

Seizures are a common feature in more than 20% of newborns with NAIS (23). In our model, two types of epileptic events were observed between ischemic onset and 24 h of reperfusion: (1) bursts of high amplitude spikes (accompanied with vocalization and myoclonic jerks) during ischemia and all along reperfusion, and (2) organized seizures consisting in discharges of a 1–2 Hz spike-and-wave only during reperfusion (from 2 up to 16 h) separated by quiet periods (24). The presence of organized seizures with a pattern close to what is observed in human newborn (23) seems related to the presence of the infarct (25). Using video-EEG and P12 rats, the first clinical and sub-clinical seizure appear during HI (27 to 53 min whatever the HI duration –75 or 90 min) (26). Unfortunately, prolonged EEG recordings and correlation with histological damages have not been performed in this study. Phenobarbital (PB) represents the first line of anticonvulsant (27), but PB only controls seizures in 50% of neonates (28). PB delayed the first seizure but did not reduced the ischemic damage in the P7 rat pup (23). More recently, flupirtine an aminopyridine first used as an analgesic, shifts the voltage to open the potassium channels to a more negative potential, was shown to be efficient when given after the first electroclinical seizure (during HI) or given after the hypoxic period in the P7 rat (29).

## Hemodynamic responses in the acute phase of ischemia

In 2011, we studied potential confounders involved in the heterogeneity of the lesion volume while surgical procedures were standardized. Our hypothesis was that the absence of cerebral lesion observed in a substantial number of animals could be partly explained by the opening of the intracranial arterial collaterality through the circle of Willis and/or through the cortical anastomoses between the vascular beds of the three terminal cerebral arteries (anterior, middle, and posterior). By the use of the non-invasive technique ultrasound (US) imaging, we were able to discriminate animals with and without detectable brain lesion (15). Indeed, whereas the CCAs are occluded during ischemia, the basilar trunk (BT) can supply all the non-occluded downstream cerebral arteries through the posterior communicating arteries. According to the mean blood flow velocity (mBFV) measured in the BT, animals with a detectable lesion do not increase their mBFV (collateral failure), whereas animals exhibiting a small lesion (or no lesion) show an increase in their mBFV (collateral recruitment—Figure 1B). A strong inverse correlation between an increase in mBFV in the BT (during ischemia) and a decrease in lesion volume at 48 h after ischemia is shown (Figure 1B), indicating the establishment of collateral supply in the neonatal P7 rat subjected to ischemia (15).

The collateral supply (established during ischemia) can also be evidenced after reperfusion on MR brain imaging. Indeed, patches and/or columnar preserved cortical areas are shown on diffusion-weighted (DW) images as early as 3 h after ischemia-reperfusion (19) and correlated with histological findings observed on cresyl violet-stained sections at 24 h, and 48 h after ischemia (Figure 1C). These columnar preserved cortical areas may be correlated with the patency of pial microvessels, as also observed in the HI model (30–32). This pattern of damage has been described in premature infants subjected to repeated events of hypoxia-acidosis associated with hypotension and is proposed to be the early pathological lesion of ulygeria (33). The magnitude of collateral supply and autoregulatory mechanisms are more often observed in immature than mature animals (34). We have investigated the effect of inhaled nitric oxide (iNO) on these mechanisms, tightly related to the collateral recruitment and brain damage extension. We demonstrated that iNO (20 ppm) is delivered to the brain, and when administered during ischemia markedly reduced the lesion size by increasing collateral recruitment (35). Conversely, the same iNO dosage appears neurotoxic when given 30 min after reperfusion, because it increases blood flow by increasing oxidative stress (accumulation of peroxynitrite—ONOO^−^) and microglial inflammation leading to an increase in the lesion size (35).

In the ischemic P7 rat pup, a progressive incomplete reperfusion is depicted during early reflow in both carotids. Consequently, this gradual reflow is correlated with reduced local cerebral blood flow (CBF) in the ipsilateral hemisphere and reduced cortical mitochondrial respiratory function (36). The no return to basal values early after reflow highlights an efficient collateral network initiated during ischemia and maintained during reperfusion. This blood flow pattern during early reflow contrasts with spontaneous arterial recanalization after CCA occlusion release observed in the juvenile rat brain subjected to the same ischemic procedure (16), and in the adult rat brain subjected to IR (37). Furthermore, whereas a significant hyperemia followed by a hypoperfusion lasting for hours is observed in the adult (38), these 2 blood-flow phases are not observed both in the neonatal (36) and juvenile rat brain (16). The absence of hyperemia during early reflow, and the lack of NO-dependent vasoreactivity in the P7 rat brain, may in part explain the inefficiency of ischemic post-conditioning after IR (39), which is in contrast reported efficient in the adult brain (40, 41) to improve ischemic damage.

## Role of NO synthases and oxidative stress

Neuronal NO synthase (nNOS) and endothelial NOS (eNOS) are endogenous mediators in CBF regulation and modulation of stroke volume after ischemia-reperfusion (42). CBF regulation is under the control of perivascular nNOS expressing structures that are colocalized with nerve terminals and mainly located in the immediate vicinity of blood vessels (43). Neuronal NOS-deficient adult mice were protected toward ischemia by the inhibition of hyperemia (see above) during early reperfusion (44), whereas nNOS-deficient neonatal mice were protected toward HI by increasing eNOS, which may counteract the reduction of CBF by the absence of nNOS (45). In our neonatal model, NO modulates blood flow during ischemia and selective inhibition of eNOS or/and nNOS has a sexual dimorphic effect on blood-flow modulation. This dual effect, however, does not strictly correlate with the resulting infarct lesion. Indeed, inhibition of nNOS induces hemodynamic changes with increased mBFVs in the BT during ischemia and reduces the lesion volume in males but not in females. In contrast, inhibition of eNOS increases mBFVs in the BT not only during ischemia but also after reperfusion leading to a detrimental effect on the ischemic lesion in males (46). This study is highlighted by the report that, in HI male rat pups, nNOS inhibition decreases peroxynitrites and reduces erythrocyte columns in the microvessels leading to a microvascular protection by restoring perfusion early after reoxygenation and providing neuroprotection (47). Peroxynitrites (ONOO^−^) and inducible NOS-immunoreactivity were measured in brain samples from rat pups after ischemia from 24 h up to 7 and 14 days of reperfusion, respectively (48), suggesting a strong oxidative stress after neonatal stroke.

## Mitochondria, cell death and sexual dimorphism

Mitochondria are intracellular organelles playing a major role in energy metabolism, generation of reactive oxygen species, and regulation of apoptosis in response to cerebral ischemia [for review see (49)]. Damage to the bioenergetic integrity of mitochondria is observed after ischemia in adult animals (50), and the formation and opening of the mitochondrial permeability transition pore (mPTP) are central mediators of this process (51–54). After ischemia, excessive calcium accumulation causes a severe reduction in mitochondrial membrane potential which triggers the formation and opening of the mPTP. Cyclosporine A inhibits the mPTP opening and reduces damage against mild ischemic injury in the P7 rat brain (55). The release of apoptogenic proteins including cytochrome c (cyt c) and apoptosis-inducing factor (AIF) is observed after opening of the mPTP and membrane permeability (56). Excessive calcium entry in the cell after ischemia leads to an important production of reactive oxygen species into the mitochondria, large mitochondrial permeability resulting in the opening of the mPTP with a subsequent release of AIF. Subsequently, an activation of poly (ADP-ribose) polymerase-1 (PARP-1), is essential to the translocation of mitochondrial AIF into the nucleus to cleave DNA, leading to a caspase-independent cell death (56). Release of cyt c may also activate caspase-3 (given cleaved caspase-3), which translocates into the nucleus to cleave PARP-1 and causes caspase-dependent cell death (57). After ischemia in P7 rat pups, males predominantly exhibit caspase-independent cell death, whereas females predominantly exhibit caspase-dependent cell death (57–59).

Caspase-dependent and -independent cell deaths are two features of cell degeneration in the developing brain after injuries including ischemia, HI and trauma with a sex dependence. Male sex is a well-recognized epidemiological risk factor for poor neurodevelopmental outcome after perinatal brain injury (60), while the mechanisms related to this sex difference remain unknown. The degree of apoptotic index with the release of cyt c in CSF after traumatic brain injury appears sex-dependent (61). Autophagy (a lysosomal pathway for intra cellular degradation of macromolecules and organelles) may also be implicated in the neonatal P7 rat brain after HI (62), and in the juvenile P12 rat subjected to focal ischemia, but only in neurons (not glial cells) (63). Although no co-occurrence of strong autophagy and caspase activation in the same neuron was found (63), it cannot be excluded that these two cell-death mechanisms act with either a different temporal profile or occur in different types of neurons. Sexual dimorphism has not been evaluated because the latter study was only investigated in male P12 rats.

## Inflammatory responses (Table 2)

Neonatal ischemia induces an inflammatory response in both the systemic circulation and parenchyma, which have however similarities and differences compared to ischemia induced in the adult brain. The establishment of inflammatory responses takes place during the energy-failure phase (second phase between 6 and 48 h) and become chronic after 48 h (third phase) in neonatal HI encephalopathy [for review see (64–66)].

**Table 2:** An overview of inflammatory responses in ischemic and hypoxic-ischemic models in the P7 rat.

**Cellular compartment and cell type**		**Stroke**			**Hypoxia-ischemia**	
		**Model**	**Responses**	**References**	**Responses**	**References**
Neurovascular unit	BBB	pMCAo+tCCAo	Early T2WI—IgG extravasation [2–72 h]	(19, 20, 72)	Early IgG extravasation (6 h) and intense extravasation at 24 h	(69)
		tfMCAo	Low Evans Blue extravasation and dextran restricted to blood vessels at 24 h—Tight junctions preserved	(67)		
	Mast cells	pMCAo+tCCAo	Increase 2–12 h with histamine Degranulation [12–48 h]	(82)	Early increase 0 to 48 h with TNF-α Cromolyn reduced damage and glial activation	(83, 84)
	Microglia	pMCAo+tCCAo	Increase in the WM [24–72 h] Increase in the cortex [72 h−2 w] and Bcl2^+^ at 72 h Ameboid [96 h−14 d] and phagocytosis	(18, 72)	Increase [4–24 h] M1 phenotype (3–24 h) M2 phenotype (24 h)	(83, 80)
		tfMCAo	Limited phagocytosis Endogenous protection (depletion of microglia exacerbates injury) Increase [24–72 h] core and penumbra	(78, 14)		
	Macrophages	pMCAo+tCCAo	24 h in the WM (migration along the corpus callosum)	(48, 72)	N.E	
		tfMCAo	Increase [4–24 h]	(14)		
	Granulocytes	pMCAo+tCCAo	From 24 h to 7 days—Peak at 72–96 h	(72)	N.E	
		tfMCAo	N.E			
Parenchyma	Astrocytes	pMCAo+tCCAo	Increase [24–48 h] Reactive [48 h−7 d] GFAP-TUNEL^+^ [6–72 h] and GFAP-Bax^+^ [14–1 m] Clasmatodendrosis [14 d−1 m]	(22, 71, 72)	Increase [4–24 h]	(83)
		tfMCAo	Reactive astrocytes [24–72 h]	(14)		

*T2WI, T2-weighted images; h, hour; d, day; m, month; w, week; N.E., Not extensively evaluated*.

The neurovascular unit or blood brain barrier (BBB) permeability after acute ischemia differs between the immature and mature rat brain, with a better-preserved integrity in the neonatal than in adult brain (67, 68). However, in our model endogenous immunoglobulin extravasation was early (2 h) found in the ipsilateral cortex in agreement with early increased signals (at 1 and 3 h after ischemic onset) observed in T2 MR brain imaging (19), commonly attributed to the development of vasogenic edematous processes due to injury of the BBB. The lack in cortical microcirculation in our model may contribute to the BBB permeability, as compared to the endovascular ischemic model used in studies described above. Endogenous immunoglobulin extravasation was also detected as early as 6 h, which became very intense at 24 h after HI insult (69). Five times higher albumin levels were measured in the cerebrospinal fluid of term neonates developing HI encephalopathy as compared to controls, highly suggesting BBB permeability (70).

Both activated endogenous (astrocyte, microglia) and infiltrating cells (macrophages/monocytes, mast cells) produce soluble inflammatory molecules such as cytokines, chemokines, reactive oxygen and nitrogen species, which are thought to be critical mediators responsible for persistent inflammation and neuronal injury. Astrocytes have not been extensively studied in the neonatal brain (71). GFAP protein increased as early as 24–48 h after ischemia in the infarcted cortex, and astrocytes become reactive, forming a dense network delineating the ischemic core (14, 72). One week after injury GFAP-positive reactive astrocytes were observed at the periphery of the lesion, and GFAP immunoreactivity, 2 weeks after injury, was characterized by fragmented processes (called clasmatodendrotic astrocytes) with apoptotic nuclei (22). Astrocyte demise participates in the ongoing deleterious process and promotes the formation of cystic lesions very similar to those observed in brain injured human newborns (22). Studies have demonstrated that GFAP measured in neonatal blood at 1–2 and 4–7 days of life were later associated with abnormal brain MRI. Similarly, GFAP in neonates with moderate-to-severe HI encephalopathy was elevated at birth and associated with abnormal neurologic outcomes (73, 74).

Resident parenchymal microglia are the primary immune cells in the brain, acting as the first defense after injury in the CNS (75). Activated microglia/macrophages were observed in the penumbra (at the periphery of the infarct) at 72 h after injury and expressed the survival promoter Bcl-2 (72), suggesting transient repair and healing as today shown with the M2 microglial phenotype. According to its environment and production of cytokines microglia can adopt an inflammatory/cytotoxic phenotype (M1-like) and/or immunomodulatory/repairing phenotype (M2-like), although this polarizing question is raised according to species, models, and brain developmental stages (immature vs. mature) (76). It is now proposed to define microglia polarization according to a disease-specific understanding based on transcriptomic and proteomic profiling (77). Lack of microglia increased levels of cytokines and chemokines already elevated by tfMCAo in the P7 rat (endovascular model—see Table 1) and increased infarct volume suggest an endogenous protective role of microglia (78). In addition, monocyte infiltration is low and the majority of macrophages in acutely injured regions are microglia (79). In our model, we observed an increased density of microglia, which progressively became ameboid and invaded the injured tissue between 96 h and 2 weeks (71). After HI in the P7 rat, an early pro-inflammatory response (polarization toward M1 phenotype) up to 24 h of recovery, followed by an anti-inflammatory response (polarization toward M2 phenotype) was reported (80).

Mast cells (MC) are normal resident cells in the CNS and are in close association with blood vessels, and regulate brain swelling and neutrophil accumulation (81). The number of MC increased between 12 and 48 h after ischemia-reperfusion in the P7 rat. Histamine immunoreactivity is detected between 2 and 12 h after reperfusion and disappears at 24 h with a concomitant MC degranulation (82). The number and activation of MC were elevated after HI in the P7 rat before cleaved caspase-3 in neurons, astroglial and/or microglial activation (83). MC stabilization by the use of cromoglicate prior HI or after HI limits the MC migration and brain damage (84). Beside these inflammatory processes, lymphocytes transiently infiltrate the white matter (corpus callosum and internal capsule) in close vicinity to blood vessels between 1 and 4 days of reperfusion (72). Neutrophils, firstly observed in arachnoid spaces and associated with intraparenchymal blood vessels at 24 h progressively invaded the upper cortical layers from 48 h up to 7 days after injury (72).

## Therapeutic interventions

Models in the immature rodents are useful to delineate cellular and/or molecular targets that may be considered as potential candidates for the protection of the developing brain. Most of these strategies target cell death (in pre-clinical settings) and inflammatory [for reviews see (64–66)] pathways.

In the 2000s several generations of caspase inhibitors have been proposed for pre-clinical studies (85). The pan-caspase boc-aspartyl-(Ome)-fluoromethyl-ketone (BAF) did not reduce the lesion size in the P7 ischemic rat, although a significant reduction in the activity of caspase-3 was measured (86). However, the animals were distributed in 2 populations (one protected and the other not protected) suggesting a sexual dimorphism, evaluation not done at that time. Using the third-generation dipeptidyl broad-spectrum caspase inhibitor quinolone-Val-Asp(Ome)-CH2-O-phenoxy (Q-VD-OPh), a significant reduction in the lesion size was observed in females but not in males at 48 h of recovery in P7 ischemic rat pups (57). In addition to genetic inhibition of caspase-2 reducing HI and excitotoxic neonatal brain injuries (87), we evaluated the effect of a specific caspase-2 inhibitor (TRP601/ORPHA 133563) in our ischemic model. A single administration of TRP601 protects against neonatal ischemia with a 6-h therapeutic time window (88) without finding a sex effect. As caspase-2 acts as an initiator caspase (before mitochondria and caspase-3 activation), this may explain why we do not have found a sexual dimorphism. As ischemic cell death may also occur via the caspase-independent pathway (DNA damage by oxidative stress, see above) this type of death involves the nuclear enzyme PARP-1, which plays a role in the repair of strand-breaks in DNA (89). Once activated by damaged DNA fragments, PARP-1 catalyzes the attachment of ADP-ribose (PAR) units to nuclear proteins, including histones and PARP-1 itself. The extensive activation of PARP-1 can rapidly lead to cell death through depletion of energy stores. Neonatal ischemia induced intranuclear accumulation of PAR (primarily in the ischemic core, and secondly in the cortical penumbra and in territories of the anterior and posterior cerebral artery) in cells preceded DNA damage (as measured with the TUNEL assay) (90). Furthermore, PARP-1 inhibition (using the non-selective PARP inhibitor 3-aminobenzamide) reduces PAR accumulation, peroxynitrites and ischemic damage, and local inflammation associated with reperfusion (91).

Melatonin, an indoleamine, is synthesized and secreted from the pineal gland relative to the circadian rhythms. Melatonin easily crosses the BBB and reaches the brain and possesses neuroprotective properties as antioxidant, free radical scavenger and modulator of the mitochondrial function (92). In the neonatal stroke model, either a single dose of melatonin 1h before ischemia or 2 doses given 1h before and 24 h after ischemia did not reduce the lesion size (93). However, melatonin was able to reduce the density of activated microglia and to promote myelinated fibers by increasing mature oligodendrocytes (93). In the HI model, melatonin given before injury and every 24 h during 6 days was able to reduce the HI lesion by inhibiting autophagy and reducing apoptosis (94). Nowadays, melatonin with its broad-spectrum antioxidant properties represents a potential therapeutic tool to improve child health [for review see (95)].

## Conclusions

Our ischemic model in the neonatal P7 rat brain, as for all others stroke models, mainly recapitulates some aspects of NAIS by targeting a limited number of cellular and/or molecular signaling, that may be considered as potential candidates as new biomarkers either in blood, urine or CSF. These cellular markers of injury can also be evaluated using non-invasive imaging assessments as cranial ultrasound (96), magnetic resonance (MR) imaging and MR angiography and/or near-infrared spectroscopy (NIRS), as recently recommended (97, 98). During these 20 years of study another important finding is that cell death, as for inflammation and oxidative stress, is sex-dependent [for review see (99)]. Optimal care may require adapted treatments according to: sex, injury course and severity, genetic risk and tissue inflammatory status. Further studies on microRNAs (miRs) regulating the microglial neuroinflammation as well as on repair, neurogenesis and regeneration should be now addressed in both sexes separately. Further development of new drugs targeting inflammatory responses and of specific agents promoting brain repair are the next challenges to improve our capabilities to protect the developing brain.

## Author contributions

All authors listed have made a substantial, direct and intellectual contribution to the work, and approved it for publication.

### Conflict of interest statement

The authors declare that the research was conducted in the absence of any commercial or financial relationships that could be construed as a potential conflict of interest.
